# Treatment of thoracolumbar kyphosis in patients with mucopolysaccharidosis type I: results of an international consensus procedure

**DOI:** 10.1186/s13023-019-0997-5

**Published:** 2019-01-18

**Authors:** Gé-Ann Kuiper, Eveline J. Langereis, Sandra Breyer, Marco Carbone, René M. Castelein, Deborah M. Eastwood, Christophe Garin, Nathalie Guffon, Peter M. van Hasselt, Pauline Hensman, Simon A. Jones, Vladimir Kenis, Moyo Kruyt, Johanna H. van der Lee, William G. Mackenzie, Paul J. Orchard, Neil Oxborrow, Rossella Parini, Amy Robinson, Elke Schubert Hjalmarsson, Klane K. White, Frits A. Wijburg

**Affiliations:** 1 Amsterdam UMC, University of Amsterdam, Pediatric Metabolic Diseases, Emma Children’s Hospital and Amsterdam Lysosome Center “Sphinx”, Meibergdreef 9, Amsterdam, Netherlands; 2 Department of Pediatric Orthopedics, Altonaer Children’s Hospital, Bleickenallee 38, 22763 Hamburg, Germany; 3 Institute for Maternal and Child Health IRCCS “Burlo Garofolo”, Trieste, Italy; 4 Department of Orthopedic Surgery, University Medical Center Utrecht, P.O. Box 85500, 3508 GA Utrecht, The Netherlands; 5 Department of Orthopaedic Surgery, Great Ormond Street Hospital for Children, London, WC1N 3JH United Kingdom; 6 Department of Paediatric Orthopaedics, Hôpital Femme-Mère-Enfant, Université Lyon 1, 69500 Lyon, Bron France; 7 Centre de Référence des Maladies Héréditaires du Métabolisme, Hôpital Femme Mère Enfant, 69500 Lyon, Bron France; 8 Department of Metabolic Diseases, Wilhelmina Children’s Hospital, University Medical Center Utrecht, P.O. Box 85090, 3508, AB Utrecht, the Netherlands; 9 Willink Biochemicals Genetics Unit, St Mary’s Hospital, Manchester University NHS Foundation Trust, Oxford Road, Manchester, M13 9WL United Kingdom; 10 Department of Foot and Ankle Surgery, Neuroorthopaedics and Skeletal dysplasias, The H. Turner institute for Children’s Orthopedics, Saint-Petersburg, Russia; 11 Amsterdam UMC, University of Amsterdam, Pediatric Clinical Research Office, Meibergdreef 9, Amsterdam, Netherlands; 12 Nemours/Alfred I. duPont Hospital for Children, Wilmington, DE USA; 13 Division of Blood and Marrow Transplantation, Department of Pediatrics, University of Minnesota, Minneapolis, MN USA; 14 Royal Manchester Children’s Hospital, Oxford Road, Manchester, M13 9WL United Kingdom; 15 Rare Metabolic Diseases Unit, Paediatric Clinic, MBBM Foundation, San Gerardo University Hospital, Via Pergolesi 33, 20900 Monza, Italy; 16 Department of Physiotherapy, Queen Silvia’s Children’s Hospital, Rondvägen 10, 416 85 Göteborg, Sweden; 17 Seattle Children’s Hospital, 4800 Sand Point Way NE, Seattle, WA 98105 USA

**Keywords:** (3–10): Mucopolysaccharidosis type I, Thoracolumbar kyphosis, Clinical practice guideline, Surgery, Brace, Dysostosis multiplex, Residual disease, International consensus meeting, Modified Delphi method, Literature review, Kyphotic angle

## Abstract

**Background:**

In all patients with mucopolysaccharidosis type I (MPS I), skeletal disease (dysostosis multiplex) is a prominent, debilitating, condition related complication that may impact strongly on activities of daily living. Unfortunately, it is not alleviated by treatment with hematopoietic cell transplantation (HCT) or enzyme replacement therapy (ERT). Although early kyphosis is one of the key features of dysostosis multiplex, there is no international consensus on the optimal management. Therefore, an international consensus procedure was organized with the aim to develop the first clinical practice guideline for the management of thoracolumbar kyphosis in MPS I patients.

**Methods:**

A literature review was conducted to identify all available information about kyphosis and related surgery in MPS I patients. Subsequently, a modified Delphi procedure was used to develop consensus statements. The expert panel included 10 spinal orthopedic surgeons, 6 pediatricians and 3 physiotherapists, all experienced in MPS I. The procedure consisted of 2 written rounds, a face-to-face meeting and a final written round. The first 2 rounds contained case histories, general questions and draft statements. During the face-to-face meeting consensus statements were developed. In the final round, the panel had the opportunity to anonymously express their opinion about the proposed statements.

**Results:**

Eighteen case series and case reports were retrieved from literature reporting on different surgical approaches and timing of thoracolumbar kyphosis surgery in MPS I. During the face-to-face meeting 16 statements were discussed and revised. Consensus was reached on all statements.

**Conclusion:**

This international consensus procedure resulted in the first clinical practice guideline for the management of thoracolumbar kyphosis in MPS I patients, focusing on the goals and timing of surgery, as well as the optimal surgical approach, the utility of bracing and required additional assessments (e.g. radiographs). Most importantly, it was concluded that the decision for surgery depends not only on the kyphotic angle, but also on additional factors such as the progression of the deformity and its flexibility, the presence of symptoms, growth potential and comorbidities. The eventual goal of treatment is the maintenance or improvement of quality of life. Further international collaborative research related to long-term outcome of kyphosis surgery in MPS I is essential as prognostic information is lacking.

**Electronic supplementary material:**

The online version of this article (10.1186/s13023-019-0997-5) contains supplementary material, which is available to authorized users.

## Background

Mucopolysaccharidosis type I (MPS I) is a rare lysosomal storage disorder caused by deficiency of the enzyme alpha-L-iduronidase (IDUA) which is involved in the degradation of the glycosaminoglycans (GAGs) dermatan sulphate and heparan sulphate and has an estimated birth incidence of 1 in every 100.000 live births [1, 2]. The clinical spectrum comprises a neuronopathic- and a non-neuronopathic phenotype [3]. The former is characterized by progressive neurodegeneration with progressive cognitive and motor impairment and comprises all Hurler patients and the severe Hurler-Scheie patients. The non-neuronopathic phenotype comprises the attenuated Hurler-Scheie patients and the Scheie patients [1]. Clinical manifestations vary in severity and age of onset but are observed in both phenotypes. The eyes, ears, airway, heart, respiratory system and the skeletal system are all commonly affected [3].

Skeletal manifestations in MPS I, collectively referred to as dysostosis multiplex, are often present at birth and are progressive. Findings include shortened long bones, short and wide clavicles, wide oarshaped ribs, odontoid hypoplasia, anterior beaking of the lower thoracic and upper lumbar vertebral bodies with secondary thoracolumbar kyphosis, bullet shaped phalanges, dysplastic femoral heads, coxa valga and genu valgum [4, 5]. Its pathophysiology has not yet been fully elucidated but probably includes inflammation, disturbed endochondral ossification and disruptions in the growth plate and articular cartilage induced directly or indirectly by intra- and extra cellular accumulation of GAGs [4, 6–9].

Disease modifying treatments of MPS I include enzyme replacement therapy (ERT) which can effectively treat several of the somatic symptoms, especially in patients with the non-neuronopathic phenotype, and hematopoietic cell transplantation (HCT) for patients with the neuronopathic phenotype, which in addition to the above has the potential to prevent or halt the central nervous system disease [10–12]. However, as the skeletal disease appears to be poorly responsive to HCT and ERT, optimizing symptomatic treatment of the skeletal manifestations is essential (i.e. analgesics, surgery) [5, 10, 13].

With increased life expectancy, progressive thoracolumbar kyphosis is now a clinically relevant skeletal complication in MPS I patients with a reported prevalence of 70–80% [14, 15]. It is associated with hypoplasia of the vertebrae and anterior wedging and retrolisthesis of the vertebral body at the apex of the curve [6, 16, 17]. It is highly likely that skeletal status significantly impacts on activities of daily living. Indeed, a qualitative study performed by the Manchester group revealed that a substantial burden is associated with musculoskeletal disease in MPS I [18]. While spinal surgery may effectively treat the thoracolumbar kyphosis in MPS I either by an anterior, posterior or a combined spinal fusion [19], there is no evidence as to how optimal treatment of kyphosis can best be achieved in MPS I patients, nor have any guidelines been published related to observation or intervention. We therefore initiated a MPS I consensus procedure by using a modified Delphi approach, with the aim to provide consensus based statements regarding the optimal treatment of thoracolumbar kyphosis in MPS I patients.

## Methods

A modified Delphi procedure was used to develop consensus statements, if possible based on evidence, otherwise on experts’ opinions, experience and intuitive judgement, as the Delphi method acknowledges the importance of this relatively subjective data in the absence of hard scientific data [20].

The steering committee (NO, SJ, FAW and GK) was formed by the principal researcher (FAW) and the process was supervised by a clinical epidemiologist (JvL). Ten spinal orthopedic surgeons (SB, MC, RC, DE, CG, VK, MK, WM, NO, and KW), 6 pediatricians (NG, PvH, SJ, PO, RP, FW) and 3 physiotherapists (PH, ESH and AR), all experienced in the treatment and follow-up of patients with MPS I, were invited to participate in this modified Delphi procedure.

To initiate the procedure a literature review was conducted by one of the researchers (GK) to evaluate the current evidence for the benefit of kyphosis surgery in MPS I (all phenotypes). The search strategy (Table 1) was developed with the aid of a clinical librarian and performed in Embase and Medline. Studies were included when kyphosis surgery in MPS I patients was reported, with at least the type of surgery and one of the following parameters: age at surgery, kyphotic angle pre-operative or levels of fusion. The results of the literature review were sent to the participants prior to the face- to-face meeting and were presented during the meeting as well.

**Table 1:** Literature search

Database	Search strategy
Medline	((exp Mucopolysaccharidosis I/) or (MPS1 or MPSI or mps-1 or mps-I or ((mucopolysaccharidos* adj (1 or I)) or ((hurler-scheie or hurler or scheie) adj (syndrom* or diseas*)))).ti,ab,kf.) AND ((exp Kyphosis/ or exp. Thoracic Vertebrae/ or exp. Spinal Cord/ or exp. Lumbar Vertebrae/) or (cobb or kypho* or vertebr* or thoracolumb* or spine or spinal or lumba*).ti,ab,kf.)
Embase	((hurler syndrome/ or scheie syndrome/) or ((mucopolysaccharidos* adj (1 or I)) or ((hurler-scheie or hurler or scheie) adj (syndrom* or diseas*))).ti,ab,kw) AND (exp Kyphosis/ or exp. spine/ or (cobb or kypho* or vertebr* or thoracolumb* or spine or spinal or lumba*).ti,ab,kw)

The Delphi procedure consisted of 3 written rounds (Additional files 1 and 2) and a face-to-face meeting. The face-to-face meeting took place on May 25, 2018 and was chaired by an independent moderator (JvL). The draft statements (Additional file 3) were presented, discussed and revised individually until full consensus was reached. Shortly after the meeting, all participants had the opportunity to anonymously express their opinion about the proposed statements in a third written round. Participants were asked whether they agreed with the statements with the following response options: agree, disagree, this is not my expertise. In case participants did not agree, a detailed explanation was mandated. The answer “this is not my expertise” was accepted (used e.g. by non-surgeons when the question was about surgical technique).

## Results

The literature review yielded a total of 18 articles related to kyphosis surgery in MPS I patients (Table 2). Only eight of these papers reported ≥2 patients. Of the 18 articles, individual patient data was extracted from 47 patients (Table 3). The median reported age at surgery was 6.4 years (range 2.4–16.8 years) and the median kyphotic angle prior to surgery was 67° (range 30° - 110°). Twenty-eight patients underwent a combined anterior and posterior surgical approach, 13 patients a posterior only approach, one patient an anterior only approach, in one patient a Vertical Expandable Prosthetic Titanium Rib technique (VEPTR) was used and in 4 patients the approach was not reported. Spinal complications after surgery were an adjacent kyphosis (*n* = 4) and an adjacent segment listhesis (*n* = 3) after a median follow-up of 3.5 years (range 1.1–8.3 years). Data on the long-term course of the kyphotic angle or on functional outcomes (i.e. activities of daily living (ADL)) and quality of life were absent in all of the reviewed publications.

**Table 2:** Summary of included studies regarding kyphosis surgery in MPS I patients

Study	Design	Patients for surgery	Age at surgery (years)	Surgical indication	Surgery type	Apex	Levels of fusion	Kyphotic angle pre- / post-operative	Complications	Neuro-physiologic monitoring	Brace	Follow-up (years)
Abelin Genevois, *JIMD,* 2014 [17]	Case series	*N* = 13 Hurler Treated between 2003 and 2010	Median age: 8 (range 3.5–15)	Deformity progression and disruption spinal balance	Single- stage circumferential arthrodesis. Combined anterior and posterior approach (*n* = 12/13)	Not reported (NR)	Two levels above and 2 levels below the apex (n = 12)	Median 60° (range 30°-90°) / Median 13° (range − 14°-52°)	Significant loss of correction (*n* = 2) ➔ revision surgery (consecutively to a previous posterior-only fusion) Mild adjacent segment spondylolisthesis at the upper level in 50%.	Multi-modal EP or at least SSEP	Postoperatively a spine cast for 3 months, in addition full time bracing for 3 months	Median 2.2 (range 0.8–8.7)
Roberts et al., *Bone Joint J*, 2016 [52]	Case series	*N* = 7 Hurler Treated between 2001 and 2013	Median 4.1 (range 2.8–16.8)	The presence of severe, progressive thoracolumbar kyphosis > 40°	Circumferential arthrodesis (*n* = 3) Circumferential arthrodesis with posterior instrumentation (*n* = 4)	T12 (*n* = 2) L1 (n = 2) L2 (*n* = 1) L1-L2 (n = 2)	T10-L3 (n = 3) T10-L4 (n = 2) T10-L5 T11-L4	Median 78° (range 56°- 110°)/ median 35° (range 0°-65°)	Deep wound infection (n = 1), stable proximal junctional kyphosis(n = 1)	SSEP and MEP	No brace	Median 5.8 (range 3.5–9.3)
Yasin et al., *Spine*, 2014 [49]	Case series	N = 7 Hurler	Anterior fusion using vascularized rib: mean 3 (range 2.4–3.8; *n* = 5) Combined anterior and posterior instrumented fusion: 8 (n = 1) VEPTR: 4 (n = 1)	NR	Anterior fusion using vascularized rib (n = 5), combined anterior and posterior instrumented fusion (n = 1), VEPTR (n = 1)	NR	NR	Anterior fusion: mean 60° (n = 5)/NR Combined fusion: 84° /at FU 35° VEPTR: 87°/ 35°	Anterior only: kyphotic deformity adjacent to the operated segment (n = 5)	NR	Postoperatively: braced until satisfactory healing of the graft on plain radiographs	NR
Tandon et al., *J Bone Joint Surg*, 1996 [30]	Case series	N = 3 Hurler	2.8 3.5 10.3	NR	Posterior spinal fusion (n = 3)	L2 (n = 2)	NR	80°/NR 56°/NR 40°/NR	At age 12y symptomatic cord compression at T10-T11 (n = 1)	NR	NR	NR
Garrido et al., *Eur Spine J*, 2014 [53]	Case series	N = 3 Hurler	2.4 2.9 3	NR	Anterior and posterior spinal arthrodesis with segmental pedicle screw instrumentation	L1 (n = 2), Th12(n = 1)	T10-L3	70°/NR 65°/NR 63°/NR	Left lower lobe collapse after extubation (n = 1) Wound infection (n = 1)	SSEP	Postoperatively: Moulded Thoracolumbar Sacral Orthosis for 3 months	NR
Vellodi et al., *Archives of Disease in childhood*, 1997 [54]	Case series	N = 7 Hurler	Average age of 7.6 (3.8–10.3) (*n* = 6)		Prophylactic posterior spinal fusion (n = 6) Thoracolumbar decompression and fusion for cord compression (n = 1)	NR	NR	NR	NR	NR	NR	NR
Field et al., *J Bone Joint Surg*, 1994 [6]	Case series	N = 6 Hurler	Average age of 7.6 (3.8 to 10.3)	NR	Posterior spinal fusion (n = 6)	L1 or L2	NR	NR	NR	NR	NR	NR
Polgreen et al., *Bone Marrow Transplantation*, 2009 [55]	Case series	N = 5 Hurler	Reported in one patient: 5	NR	Spinal fusion (n = 2), anterioposterior spinal fusion (n = 1), posterior spinal fusion (n = 2), anterior spinal fusion (n = 1)	NR	T9 – L3 T10-L2 T11 - L3 T7 -L3 1 not reported	NR	NR	NR	Adjacent kyphosis to operated segment T1-T8	NR
Schmidt et al., *Orphanet Journal of Rare diseases,* 2016 [33]	Case series	N = 2 Hurler	7.8 14.3	Symptoms, not further specified (n = 1)	Posterior fusion (n = 2)	NR	NR	90°/ NR (n = 1)	NR	NR	No	NR
Hopwood et al.*, JIMD*, 1993 [56]	Case series	N = 2 Hurler	7 10.3	NR	Posterior spinal fusion (n = 1) Posterior spinal fusion using the patient’s own left anterior iliac spine plus donor bone. (n = 1)	NR	NR	NR	NR	NR	NR	NR
Malm et al., *Acta Paediatrica*, 2008 [57]	Case series	N = 2 Hurler	NR	NR	Spinal fusion Dorsal spinal fusion and ventral spinal fusion	NR	Spinal fusion: T8 and L4 Dorsal spinal fusion: T10 to L4 and ventral spi- nal fusion: T12 - L3	NR	NR	NR	NR	NR
Souillet et al., *Bone marrow transplantation*, 2003 [58]	Case series	N = 2 Hurler	11.2 and 12.2 3.4	NR	Posterior spinal fusion and anterior spinal fusion (n = 1) Posterior spinal fusion (n = 1)	NR	NR	NR	NR	NR	No	NR
Stoop et al., *JIMD reports,* 2012 [45]	Case series	N = 1 Hurler Treated between 2003 and 2011	NR	NR	Short segment posterior fusion after pedicle subtraction osteotomy of L1	NR	Th12 to L3	80°/ 12°	Kyphosis superior to the spondylodesis; 64°➔ revision of the spondylodesis at the level of T11-L4 and superior extension with a growing rod system; 14°	NR	No.	NR
Yasuda et al., *MGM reports*, 2015 [59]	Case report	N = 1 Hurler	13	NR	Arthrodesis. Thoracolumbar spinal fusion surgery, dual fusion rods with pedicle screws extended from T5- L3 with overlying graft material	L2	T5-L3	NR	NR	NR	NR	NR
Bekmez et al., *J Pediatr Orthop*,2016 [43]	Case report	N = 1 Hurler Treated between 2008 and 2011	8	Deformity progression of > 10 °/ 12 months and disruption of the sagittal balance despite brace treatment	Posterior only	L1	T10- L3	52°/ 15°	Distal junctional Kyphosis of 18 °	SSEP and MEP	Postoperatively, thoracolumbar sacral orthosis, for 4 months.	5.1
Makler et al., *Surg Neurol Int*, 2017 [60]	Case report	N = 1 Hurler	6	NR	Anterior release and posterior spinal fusion	L1	NR	Complete resolution of gibbus deformity	Preoperative work up revealed a Chiari I malformation and a syringomelia	NR	NR	NR
Dalvie et al., *Spine*,2001 [44]	Case report	N = 1 Hurler	5	NR	Anterior instrumented correction and fusion	NR	T11-L2	59°/18°	NR	NR	NR	0.4
Pauchard et al., *JIMD reports,* 2014 [61]	Case report	N = 1	4	NR	Two-stage surgical correction was planned, with initial posterior instrumentation of T12-L2, followed by anterior graft. After first surgery a complication ➔leading to removal of the hardware.	T12	T12-L2	90°/NA	Asymmetric paraplegia with partial sensory loss	SSEP and MEP	NR	NR

*Abbreviations*: *VEPTR* Vertical expandable prosthetic titanium rib, *SSEP* somatosensory- evoked potentials, *MEP* motor evoked potentials

**Table 3:** Individual MPS I patients – kyphosis surgery

Study	Patient ID (as reported in article)	Age at surgery (years)	Apex	Surgical approach	Levels of fusion	Kyphotic angle Preoperative/postoperative	Complications	Angle at follow-up	Follow-up (years)
Abelin Genevois, *JIMD,* 2014 [17]	1	8.5	L1	Anteroposterior (circumferential) arthrodesis	T10-L4; corp T12-L2	70°/30°	Sylvian infarction	−29°	1
Abelin Genevois, *JIMD,* 2014 [17]	2	12	L1 L2	Posterior only arthrodesis; Anteroposterior (circumferential) arthrodesis	T11-L3	60°/13°	Adjacent segment listhesis: revision	21°	1.9
Abelin Genevois, *JIMD,* 2014 [17]	3	7.5	L2 L3	Anteroposterior (circumferential) arthrodesis	T11-L4	90°/−	Cardio-respiratory failure	Not reported (NR)	NR
Abelin Genevois, *JIMD,* 2014 [17]	4	8	L1 L2	Anteroposterior (circumferential) arthrodesis	T9-L3; corp T11-T12	50°/6°	Adjacent segment listhesis	39°	8.3
Abelin Genevois, *JIMD,* 2014 [17]	5	3.5	L1	Posterior only arthrodesis; Anteroposterior (circumferential) arthrodesis	T12-L5; corp L2-L3	90°/−	Adjacent segment listhesis: hip luxation	NR	3.5
Abelin Genevois, *JIMD,* 2014 [17]	6	15	L2 L3	Anteroposterior (circumferential) arthrodesis	T12-L5; corp L2-L3	65°/45°	Transient paraparesis	NR	2
Abelin Genevois, *JIMD,* 2014 [17]	7	9	L2	Anteroposterior (circumferential) arthrodesis	T11-L1; corp T1	45°/10°	No	20°	1.8
Abelin Genevois, *JIMD,* 2014 [17]	8	6	L3	Anteroposterior (circumferential) arthrodesis	L1-L4; corp L2-L3	30°/−4°	No	−10°	7.2
Abelin Genevois, *JIMD,* 2014 [17]	9	4.5	T12	Anteroposterior (circumferential) arthrodesis	T12-L3	84°/42°	Progressive scoliosis	−28°	0.9
Abelin Genevois, *JIMD,* 2014 [17]	10	9	L2	Anteroposterior (circumferential) arthrodesis	L1-L3; corp L2	35°/13°	No	21°	2.4
Abelin Genevois, *JIMD,* 2014 [17]	11	8.5	L2	Anteroposterior (circumferential) arthrodesis	T12-L3; corp L2	30°/−14°	No	25°	0.8
Abelin Genevois, *JIMD,* 2014 [17]	12	5	L2	Anteroposterior (circumferential) arthrodesis	T11-L3; corp T12-L2	34°/−10°	No	25°	6.3
Abelin Genevois, *JIMD,* 2014 [17]	13	5	L2	Anteroposterior (circumferential) arthrodesis	T12-L3	70°/52°	Hip dislocation	−22°	8.7
Roberts et al., *Bone Joint J*, 2016 [52]	7	6.8	T12	Circumferential arthrodesis	T10-L3	110°/65°	No	NR	7.2
Roberts et al., *Bone Joint J*, 2016 [52]	8	7.7	L1	Circumferential arthrodesis	T10-L4	94°/65°	No	NR	9.3
Roberts et al., *Bone Joint J*, 2016 [52]	9	16.8	L1	Circumferential arthrodesis with posterior instrumentation	T10-L5	78°/35°	No	NR	4.6
Roberts et al., *Bone Joint J*, 2016 [52]	10	2.8	L2	Circumferential arthrodesis with posterior instrumentation	T11-L4	56°/0°	Deep wound infection; stable 3.5proximal junctional kyphosis	NR	3.5
Roberts et al., *Bone Joint J*, 2016 [52]	11	4.1	L1-L2	Circumferential arthrodesis	T10-L4	110°/55°	No	NR	6.6
Roberts et al., *Bone Joint J*, 2016 [52]	12	3.1	T12	Circumferential arthrodesis with posterior instrumentation	T10-L3	62°/22°	No	NR	5.1
Roberts et al., *Bone Joint J*, 2016 [52]	13	3.4	L1-L2	Circumferential arthrodesis with posterior instrumentation	T12-L2	69°/12°	No	NR	5.8
Yasin et al., *Spine*, 2014		10		Combined anterior and posterior instrumented fusion	NR	84°	NR	36°	0.5
Yasin et al., *Spine*, 2014		4		VEPTR	NR	87°	NR	35°	1
Tandon et al., *J Bone Joint Surg*, 1996 [30]	1	2.8	L2	Posterior spinal fusion	L1-L3	80°/NR	NR	NR	NR
Tandon et al., *J Bone Joint Surg*, 1996 [30]	5	10.3	L2	Posterior spinal fusion	NR	56°/NR	NR	NR	NR
Tandon et al., *J Bone Joint Surg*, 1996 [30]	7	3.5	NR	Posterior spinal fusion	NR	40°/NR	At age 12y symptomatic cord compression at T10-T11	NR	NR
Garrido et al., *Eur Spine J*, 2014 [53]	2	2.4	L1	Anterior and posterior spinal arthrodesis with segmental pedicle screw instrumentation	T10-L3	70°/ 13° at follow up	Left lower lobe collapse after extubation	13°	NR
Garrido et al., *Eur Spine J*, 2014 [53]	3	3	T12	Anterior and posterior spinal arthrodesis with segmental pedicle screw instrumentation	T10-L3	63°/13° at follow up	No	13°	NR
Garrido et al., *Eur Spine J*, 2014 [53]	4	2.9	L1	Anterior and posterior spinal arthrodesis with segmental pedicle screw instrumentation	T10-L3	65°/12° at follow up	Wound-infection	12°	NR
Polgreen et al., *Bone Marrow Transplantation*, 2009 [55]	3	NR	NR	Spinal fusion	T9- L3	NR	NR	NR	NR
Polgreen et al., *Bone Marrow Transplantation*, 2009 [55]	5	NR	NR	Anteroposterior fusion	T10-L2	NR	NR	NR	NR
Polgreen et al., *Bone Marrow Transplantation*, 2009 [55]	6	5	NR	Posterior spinal fusion	NR	NR	Progression of kyphosis adjacent to previous fusion T1-T8	NR	NR
Polgreen et al., *Bone Marrow Transplantation*, 2009 [55]	7	NR	NR	Spinal fusion	T11-L3	NR	NR	NR	NR
Polgreen et al., *Bone Marrow Transplantation*, 2009 [55]	8	NR	NR	Posterior spinal fusion	T7-L3	NR	NR	NR	NR
Schmidt et al., *Orphanet Journal of Rare diseases,* 2016 [33]	15	7.8	NR	Posterior fusion	NR	90/NR	NR	NR	NR
Schmidt et al., *Orphanet Journal of Rare diseases,* 2016 [33]	19	14.3	NR	Posterior fusion	NR	NR	NR	NR	NR
Malm et al., *Acta Paediatrica*, 2008 [57]	A	NR	NR	Spinal fusion between T8 and L4	NR	NR	NR	NR	NR
Malm et al., *Acta Paediatrica*, 2008 [57]	B	NR	NR	Posterior spinal fusion of T10 to L4 and ventral spinal fusion of T12 to L3	NR	NR	NR	NR	NR
Hopwood et al.*, JIMD*, 1993 [56]	P.F.	7	NR	Posterior spinal fusion	NR	NR	NR	NR	NR
Hopwood et al.*, JIMD*, 1993 [56]	E.H.	10.3	NR	Posterior spinal fusion using the patient’s own left anterior iliac spine plus donor bone	NR	NR	NR	NR	NR
Souillet et al., *Bone marrow transplantation*, 2003 [58]	1	11.2 and 12.2	NR	Posterior spinal fusion and anterior spinal fusion	NR	NR	NR	NR	NR
Souillet et al., *Bone marrow transplantation*, 2003 [58]	12	3.4	NR	Posterior spinal fusion	NR	NR	NR	NR	NR
Stoop et al., *JIMD reports,* 2012 [45]	5	NR	NR	Short segment posterior fusion after pedicle subtraction osteotomy of L1	Th12 - L3	80°/ 12°	Kyphosis superior to the spondylodesis: 64°	NR	1.1
Yasuda et al., *MGM reports*, 2015 [59]	–	13	L2	Spinal fusion	T5-L3	NR	NR	NR	NR
Bekmez et al., *J Pediatr Orthop*,2016 [43]	3	8	L1	Posterior fusion	T10-L3	52°/15°	Distal junctional kyphosis	17	5.1
Makler et al., *Surg Neurol Int*, 2017 [60]	–	6	L1	Anterior release and posterior spinal fusion	NR	NR	NR	Complete resolution	3
Dalvie et al., *Spine*,2001 [44]	7	5	NR	Anterior instrumented correction and fusion	T11-L2	59°/18°	No	NR	0.4
Pauchard et al., *JIMD reports,* 2014 [61]	2	4	T12	Planned two-stage surgical correction, with initial posterior instrumentation of T12-L2, followed by anterior graft. After posterior surgery a complication➔ leading to removal of the hardware	T12-L2	90°/NR	Asymmetric paraplegia with partial sensory loss, requiring emergency surgery to remove the hardware	NR	NR

All of the invited experts participated in at least one round (Table 4). During this Delphi procedure, sixteen statements were developed and full consensus was reached on all statements.

**Table 4:** Number of participants Delphi Procedure

Round	Written round 1	Written round 2	Face-to-face meeting	Written round 3
Number of participants	18	17	16	19

### Statement 1

18 participants agreed and 1 stated: “this is not my expertise”

*The aim of kyphosis surgery in MPS I patients is correction and prevention of ongoing progression of kyphosis with a satisfactory neurological, biomechanical (*i.e. *improvement of sagittal balance) and cosmetic outcome for the patient, with maintenance or improvement of activities of daily living.*

The immediate goal of surgery is correcting the kyphotic deformity and preventing recurrence and progression of the deformity. The ultimate goal is to maintain the ADL in patients with an asymptomatic kyphosis as performance may deteriorate in the future or, in patients who have clinical symptoms due to their kyphosis, such as back pain, sagittal imbalance or neurological impairment, to improve daily life performance. Furthermore, correction of the kyphosis can lead to an improvement of the patient’s posture, thus improving cosmetic appearance, which may benefit the quality of life of older patients with relatively intact cognition.

As data on long-term outcomes is lacking, future studies are warranted to address these, especially focused on quality of life and daily life performance [21].

More data is available on surgical intervention in non-MPS related kyphosis. Murray et al. reported that in adult patients with Scheuermann kyphosis (average age 35 years, range 25–82) increasing curve magnitude led to more concern about their appearance compared to a healthy control group. Also, patients with the apex at a caudal level seemed to have less self-esteem issues with increasing magnitude of the kyphotic deformity [22]. Quality of life including physical functioning improved after corrective spinal surgery in adult patients with a symptomatic thoracolumbar or lumbar kyphosis secondary to osteoporosis [23]. Patients with Scheuermann kyphosis showed that functional outcome significantly improved 2 to 8 years after surgery. However, long term follow-up (14 to 21 years postoperatively) of the same patient cohort showed no difference in functional outcome compared to the preoperative scores [24]. However, the nature of the MPS disease, the age group, comorbidities and intellectual function strongly limit comparison with Scheuermann kyphosis.

Despite the fact that respiratory improvement after kyphosis correction has been reported in patients with Scheurmann kyphosis and kyphosis due to severe osteoporosis, this was not included in the statement since the panelists agreed that their collective experience with MPS I did not support this.

### Statement 2

17 participants agreed and 2 stated: “this is not my expertise”

*The timing of surgery depends on the progression and flexibility of the spinal deformity, the presence of symptoms, growth potential and comorbidities.*

*If indicated, surgery is generally performed between 5 and 13 years of age.*

While it was agreed that timing of surgery is not based on age, it was noted that kyphosis surgery at a very young age may have a higher risk of implant failure. Furthermore, at a very young age, HCT complications may adversely affect the health of the patient and their ability to withstand surgery safely. In older patients, cardiac involvement increases the risks of surgery [25, 26]. It was also agreed that kyphosis surgery in older patients may be made more complicated by increasing stiffness of the deformity. As a result, it was concluded that patients are generally operated on between 5 and 13 years of age (Table 2) with the exception of extreme kyphosis and/or neurological signs and symptoms in very young patients or mature patients.

### Statement 3

18 participants agreed and 1 stated: “this is not my expertise”

*The indication for surgery should be made by a multidisciplinary team, including the spinal surgeon. The spinal surgeon finalizes the decision in consensus with the patient/parents.*

Because of the complexities involved in the treatment of patients with MPS I, a multi-disciplinary team (MDT) needs to be involved. Such an MDT will include a metabolic specialist or clinical geneticist (often also acting as the coordinator of the team), an orthopedic and spinal surgeon, a physiotherapist, a rehabilitation specialist, an occupational therapist, a cardiologist, an anesthesiologist, an ear nose and throat specialist and a pulmonologist, all preferably with experience in MPS [17, 27–29].

An MPS I patient with a kyphosis is referred to a spinal surgeon who initially decides on the indication for surgery, which will first be discussed with the metabolic specialist and with the patients/parents. Before the final decision is made, the MDT is essential to assess the risk of potential complications and contraindications. The organization of an MDT will differ between centers and countries, and different strategies for consultation and communication can be used effectively. It is essential that a case manager/coordinator is appointed.

### Statement 4

17 participants agreed and 2 stated: “this is not my expertise”

*Preoperative full spine and brain MRI should be evaluated for spinal cord compression at sites away from the kyphotic deformity, particularly the occipito-cervical junction and the cervico-thoracic junction.*

While spinal and brain MRIs are recommended every other year in MPS I patients [27], it is essential to assess the spinal column and brain by MRI prior to kyphosis surgery for the presence and degree of spinal stenosis and cord compression at sites away from the deformity, as well as hydrocephalus. Furthermore, plain lateral cervical X-ray studies, if feasible in voluntary flexion and extension, are necessary to detect the presence of atlantoaxial instability, which is reported in 20% of transplanted Hurler patients [14]. This information is needed to prevent neurological complications during and immediately after surgery.

### Statement 5

18 participants agreed and 1 stated: “this is not my expertise”

*For patients presenting with back pain, it is essential to first explore the cause of the pain and to try non-operative therapeutic options (*e.g. *physiotherapy, pain medication and/or a brace). Surgery may be considered after all other options to treat the pain have failed, which is considered a rare event.*

Back pain as a single symptom is rarely an indication for surgery. There are a number of possible causes for back pain in MPS I, such as problems related to the intervertebral discs, muscles, ligaments, facet joints and back pain as a result of posture deviations due to hip problems. To provide adequate non-operative treatment it is important to determine the cause. When non-operative management is ineffective and back pain is severe, surgery of spine deformities may be an option.

With increasing life expectancy, due to improvement of supportive care, symptomatic treatment and treatment with HCT, patients may suffer from sagittal imbalance for a considerable time which may lead to an increase in the number of patients presenting with back pain.

### Statement 6A

17 participants agreed and 2 stated: “this is not my expertise”

*Abnormal clinical neurological signs and symptoms caused by kyphosis (though rare) are an indication for kyphosis surgery.* Only a few MPS I patients with neurological signs and symptoms, attributable to a thoracolumbar kyphosis have been reported [13, 30]. Despite its rarity, it is generally agreed that MPS I patients with a thoracolumbar kyphosis and abnormal clinical neurological signs and symptoms should undergo surgery.

### Statement 6B

17 participants agreed and 2 stated: “this is not my expertise”

*Signs of spinal cord compromise on spinal MRI at the level of the kyphosis and/or detected by electrophysiological studies can be an indication for surgery.*

With the purpose of detecting myelopathy at an early stage, an MRI scan of the spine is recommended every other year during follow-up of MPS I patients [27]. Signs of myelopathy on MRI and/or electrophysiological studies can be present without accompanying clinical signs or symptoms, but when progressive or severe, warrant surgical decompression. Since progressive cord myelopathy is difficult to interpret in patients with MPS I, it is preferable that a radiologist with experience in MPS I should evaluate the MRI.

### Statement 7

19 participants agreed

*A low developmental quotient is not a contra-indication for surgery where a benefit in quality of life can be expected.*

Patients with MPS I (both with the Hurler and severe Hurler-Scheie phenotype) often have neurocognitive impairment despite HCT [10, 31].

Intellectual ability is often expressed as developmental quotient (DQ), defined as the developmental age divided by the chronological age multiplied by 100 [32]. There was full agreement that DQ should be taken into account when considering kyphosis surgery, but that a low DQ is not a contra-indication for surgery if a benefit in quality of life is expected by correcting the kyphosis.

### Statement 8

18 participants agreed and 1 stated: “this is not my expertise”

*Hip range of motion should be taken into account when kyphosis surgery is considered.*

Hip dysplasia is present in most Hurler patients, even after successful HCT, and may lead to a restricted hip range of motion [5, 14, 33–35]. Since both the hip and spine are involved in maintaining sagittal balance, it is important to consider the contribution of both when discussing kyphosis surgery. Compensatory mechanisms may negatively impact the outcomes of surgery (e.g. in a patient with severe hip contractures, compensatory lordosis of the lumbar spine after kyphosis surgery may lead to sagittal imbalance). In addition, knee flexion and ankle plantarflexion (though to a lesser extent) should also be taken into account since this may also impact spinal alignment.

The optimal timing of hip surgery and kyphosis surgery in a patient needs to be assessed by a hip surgeon and a spinal surgeon, in consultation with the MDT. In general, hip surgery will be performed at a relatively earlier age than spine surgery because this allows reconstructive surgery whereas at an older age a salvage procedure (e.g. a shelf augmentation or Chiari osteotomy) will be the only option [36].

### Statement 9

17 participants agreed and 2 stated: “this is not my expertise”

*Patient positioning for spinal radiographs should be standardized (preferably standing and unsupported). If this is not feasible for an individual patient the position of the patient should be reported.*

To adequately evaluate the spinal deformity, an anteroposterior and a lateral radiograph are required. The radiographs should include the total thoracolumbar spine and the pelvis, with patients preferably in the standing position focused on straight knees and hips. This is important because patients tend to compensate sagittal imbalance by thoracic lordosis, pelvic retroversion and flexion of the knees [17, 37, 38]. Furthermore, it was strongly recommended to report the patient’s position on both the radiograph and the report, since they can become disconnected.

### Statement 10

15 participants agreed and 4 stated: “this is not my expertise”

*Radiographic assessment of the kyphotic deformity in MPS I should include angular and translational measurements on serial radiographs.*

Traditionally the kyphotic angle is measured by the Cobb method [39], however due to dysplasia of the vertebrae, application of this method can be challenging as the contours of the upper endplate of the upper vertebra and of the lower endplate of the lower vertebra are often difficult to assess. It appears that clinicians use approximations applicable to the specific radiographic abnormalities. While this is generally adequate for clinical practice, for research purposes and communication, a standardized protocol stating how to measure the kyphotic angle in patients with MPS I is essential. The same applies for the translational data, i.e. the severity of spondylolisthesis. Vertebral beaking, (sub)luxation of vertebrae and scoliosis are also regularly observed in MPS I in combination with kyphosis, and need to be evaluated on radiographic examination [13, 33]. It is important to evaluate the sagittal balance which is determined by a combination of the position of the spine, hips, knees and to a lesser extent the ankles. Measuring sagittal balance on radiography can be challenging in MPS I patients, as patients need to stand still, unsupported and without bending forward.

Kyphosis surgery should be based on the evaluation of more than 1 radiograph, in order to be able to assess the extent of progression. Radiographs should be routinely performed at a minimum of every other year [27].

### Statement 11

18 participants agreed and 1 stated: “this is not my expertise”

*Neurological monitoring is mandatory during kyphosis surgery. Surgery should only be carried out in a unit with the capability to carry out appropriate multimodality neurophysiological monitoring including the anterior motor pathway.*

To reduce the risk of paralysis, it is important to monitor the function of the spinal cord pre- and peri-operatively. It was agreed that optimal monitoring includes both motor evoked potentials (MEPs) and somatosensory evoked potentials (SSEPs). MEPs are considered essential since they assess the anterolateral part of the spinal cord [40], which may be stretched during the procedure, potentially resulting in motor impairment. Obtaining MEPs can be difficult or even impossible in young (approx. < 5 years) patients due to neurophysiological immaturity of the cortical pathways. In addition, MEPs are contra-indicated in some patients with seizure disorders [41]. In those cases, medulla stimulation may be used as alternative approach to monitor these tracts [42].

### Statement 12

15 participants agreed and 4 stated: “this is not my expertise”

*The posterior only approach and the combined (anterior and posterior) approach both have benefits and disadvantages, and the decision regarding which surgical approach to use should be based on the patient’s size and weight and the degree and flexibility of deformity of the spine.*

The following advantages of the posterior only approach were raised during this session: it is less invasive and the chest is not opened, there is less morbidity, a shorter procedure, a shorter hospital stay and it is less likely that patients have significant postoperative recovery issues. The reported disadvantages of the posterior only approach were: it leads to less correction of the kyphotic angle, efficient fusion is not achievable, it leads to further destruction of the weak posterior ligamentous complex, there is a risk of a possible future need for anterior surgery and adequate fixation can be difficult in the dysplastic vertebrae. The reported advantages of the combined approach (i.e. both anterior and posterior approaches) are that it leads to better correction and stability. The reported disadvantages of the combined approach include the obvious facts that a two incision approach is more invasive, the diaphragm is taken down, there is a higher risk of pulmonary complications and morbidity, the surgery is technically demanding, the operating time is longer and an ICU admission more likely. Although, the combined approach was regarded as the optimal strategy, several experts from the panel mentioned that nowadays a preference is emerging to the posterior only approach as stronger implant systems have been introduced leading to good posterior column fixation. If satisfactory alignment cannot be achieved by a posterior approach only through posterior osteotomies [43], a subsequent anterior approach should be performed. The decision on the optimal approach in individual patients will be influenced by the patient’s size, weight, degree and flexibility of the deformity and the experience of the surgeon. In case of a large deformity and a stiff and/or large spine, an additional anterior approach may be the best option.

### Statement 13

16 participants agreed and 3 stated: “this is not my expertise”

*Selection of the number of surgical levels to be fused during kyphosis surgery depends on the degree of deformity, the number of dysplastic segments, the level of the kyphosis, the approach used and expected correction. Instrumenting at minimum 2 levels above and 2 below the dysplastic segments seems to produce adequate alignment. However, despite adequate surgical management, junctional malalignment can be a complication.*

The selection of the number of surgical levels depends on several factors, but a minimum of 2 levels above and 2 levels below the dysplastic segments is considered to be sufficient as reported in several publications [17, 44, 45]. When optimal correction cannot be achieved during surgery, another level can be added to improve correction. Unfortunately, there are no long-term follow up data on the outcome related to the levels of fusion and during the meeting several experts reported, that spines initially considered adequately corrected may show kyphotic or junctional failure and malalignment during long term follow up, this was also discussed in the article of Bekmez et al. [43].

### Statement 14

18 participants agreed and 1 stated: “this is not my expertise”

*Bracing in young children with large flexible deformities may be considered as it may postpone surgery up to an age where vertebral development allows rigid fixation.*

There is no consensus whether a brace is effective in the treatment of kyphosis. However, bracing prior to surgery may be considered in young patients with large flexible deformities as it may help to maintain sagittal balance, and may slow down the progression of the deformity and thus delay surgery until patients reach an age where surgery is expected to be technically more successful. Bracing is generally well accepted in young patients.

In patients with back pain due to kyphosis, lightweight flexible braces may be advised to offer comfort.

### Statement 15

17 participants agreed and 2 stated: “this is not my expertise”

*Bracing post-surgery may be considered for a period of 3 to 6 months as it may protect the arthrodesis and the adjacent segments.*

There are several reasons to use bracing in MPS I patients after kyphosis surgery*.* First, due to the small dysplastic vertebrae of MPS I patients, fixation may be not as strong as fixation in non-MPS I patients. Second, patients with developmental delay may be over-active and/or have problems following instructions. This may jeopardize the surgical construct. However, it should be taken into account that brace compliance in these older patients may be low because of discomfort.

### Statement 16

17 participants agreed and 2 stated: “this is not my expertise”

*Treatment algorithms based on measurement of the kyphotic angle alone are not sufficient in MPS I patients. Indication should not be based on curve magnitude only, but on the indications as discussed in statement 6.*

General indications for spinal surgery in kyphosis with another etiology cannot be applied to MPS I patients. For example, Wenger et al. proposed that patients with a Scheuermann kyphosis > 75°, or a significant kyphosis (> 65°) associated with pain that cannot be alleviated with conservative treatment, should be considered for surgery [46, 47]. Vaccaro et al. report an angle of 30° (as an additional criterion) in patients with a traumatic thoracic kyphosis be used as an indication for surgery [48]. For MPS I it has been reported, that kyphotic deformities exceeding 45 degrees tend to progress [49], and as such should be monitored more closely. In addition, progression exceeding 15 degrees per year has been proposed as an indication for surgery [43].

During the meeting, all experts agreed that no mandatory cut-off value for the Cobb angle can be established for kyphosis surgery in MPS I patients. This decision depends on the presence of abnormal clinical neurological signs and symptoms and MRI and/or electrophysiological signs of spinal cord compromise (statement 6). In addition, it also depends on the deformity progression and flexibility, the presence of symptoms, growth potential and comorbidities (statement 3).

## Discussion

This is the first clinical practice guideline for the management of kyphosis in MPS I patients. Because of the high frequency of kyphosis in MPS I [14, 15] and the increased life expectancy in MPS I due to improved results of treatment [10] there is an urgency to optimize treatment. In the absence of robust data in literature, this guideline was developed based on an international consensus procedure according to the modified Delphi method [50].

The paucity of literature is not surprising for this very rare disorder with a slow evolution of signs and symptoms, including kyphosis and its potential complications. No prospective, randomized controlled trials have been conducted and only a few high-quality case series have been published [17, 49, 51]. As these articles all focus on the timing and approaches of surgery and do not report on long term outcomes, we concluded that, in the absence of higher level of evidence, development of a clinical practice guideline can only be achieved by combining the limited available evidence with expert opinions.

Although some topics were on technical surgical details, it was a deliberate decision to also involve metabolic pediatricians and physiotherapists, since decisions on surgery in this very complex patient group need to take all aspects of the patient’s condition into account. International experts were invited in order to obtain widespread international knowledge. After 3 written rounds and a face-to-face meeting, full consensus was achieved on all statements and on the additional information and discussions on each statement.

The current study has several limitations. First, the aim of the Delphi method is to reach a convergence of opinion. To overcome the possibility that participating experts might experience pressure to conform with the group, the responses to the written rounds were not visible for other experts. The face-to-face meeting was moderated by a clinical epidemiologist experienced in chairing Delphi meetings. Finally, at the end of the procedure, the statements resulting from the face-to-face meeting were presented to all the experts inviting them to indicate whether they agreed with these statements and the answers were collected. A second limitation was that three participants were unable to attend the face-to-face meeting and thus could not contribute to the discussions and formulation of the final statements. They did, however, complete the questionnaires and we decided to include them in the final consensus statements.

This international consensus procedure resulted in a set of statements on thoracolumbar kyphosis with explanation and discussion that can be used as a clinical practice guideline for clinicians involved in the follow-up and treatment of MPS I patients. All experts agreed that more research is needed, in particular on the long-term follow-up assessing the effects of kyphosis surgery, on functional outcomes and quality of life. Furthermore, it was agreed that there is a need for a standardized protocol for the reproducible measurement of all aspects of the kyphotic deformity as this is important for research and communication.

## Conclusions

This international consensus procedure resulted in the first clinical practice guideline for the management of kyphosis in MPS I patients, focusing on the aim and the indication for surgery, the approach and timing of surgery, the additional assessments that are required and the utility of bracing. Most importantly, it was concluded that the decision for surgery not only depends on the kyphotic angle, but on several factors, including the deformity progression and flexibility, the presence of symptoms, growth potential and comorbidities and that the eventual goal of treatment is the maintenance or improvement of quality of life. Further research is needed to gain insight into the long-term outcomes after kyphosis surgery, especially focusing on the impact on ADL and on quality of life.

## Additional files

Additional file 1: Written round 1. (DOC 625 kb)

Additional file 2: Written round 2. (DOC 46 kb)

Additional file 3: Draft statements to be discussed at the face-to-face meeting. (DOC 67 kb)
