# Supplementary material for: Treatment of thoracolumbar kyphosis in patients with mucopolysaccharidosis type I: results of an international consensus procedure
Source: Orphanet J Rare Dis. 2019 Jan 18;14:17. doi: 10.1186/s13023-019-0997-5 (PMC6339313; doi:10.1186/s13023-019-0997-5)
Supplement: Supplementary file 1 — Written round 1. (DOC 625 kb) [file 13023_2019_997_MOESM1_ESM.doc]

Additional file 1: Written round 1

The first online written round contained 2 case histories and general questions with the aim of collecting as much relevant information as feasible. The case histories included patients with the Hurler phenotype with thoracolumbar kyphosis, including information regarding the age at diagnosis and HCT, and the timing of hip or knee surgery. The first patient had a progressive kyphosis with associated clinical symptoms, and the second patient had little to no progression of the kyphosis and minimal clinical symptoms. Sequential radiographs were provided, all prior to surgery. The participants were asked whether they would perform kyphosis surgery in these patients, what factors influenced their decision, what the aim of surgery would be, and what timing and approach they would prefer. The cases and questions were composed by the steering committee (FAW, GK, SJ, NO) and the clinical epidemiologist (JvL).

**Case 1: Patient A. (age 11.5 years)**

- Male, diagnosis Hurler at 12 months
- HCT at 14 months
- Clinical course:
  - Cobb angle progressed from 44.9 degrees at age 2 to 91 degrees at age 11.5 years
  - Knee surgery (8 plates) at age 11 years
- Symptoms
  - Daily complaints about backpain
  - Difficulties in bending forward
  - Difficulties with standing up from sitting position
  - Seems to stumble more often
- Neurological examination: no abnormalities
- MRI: no spinal cord compression
- Radiographs:


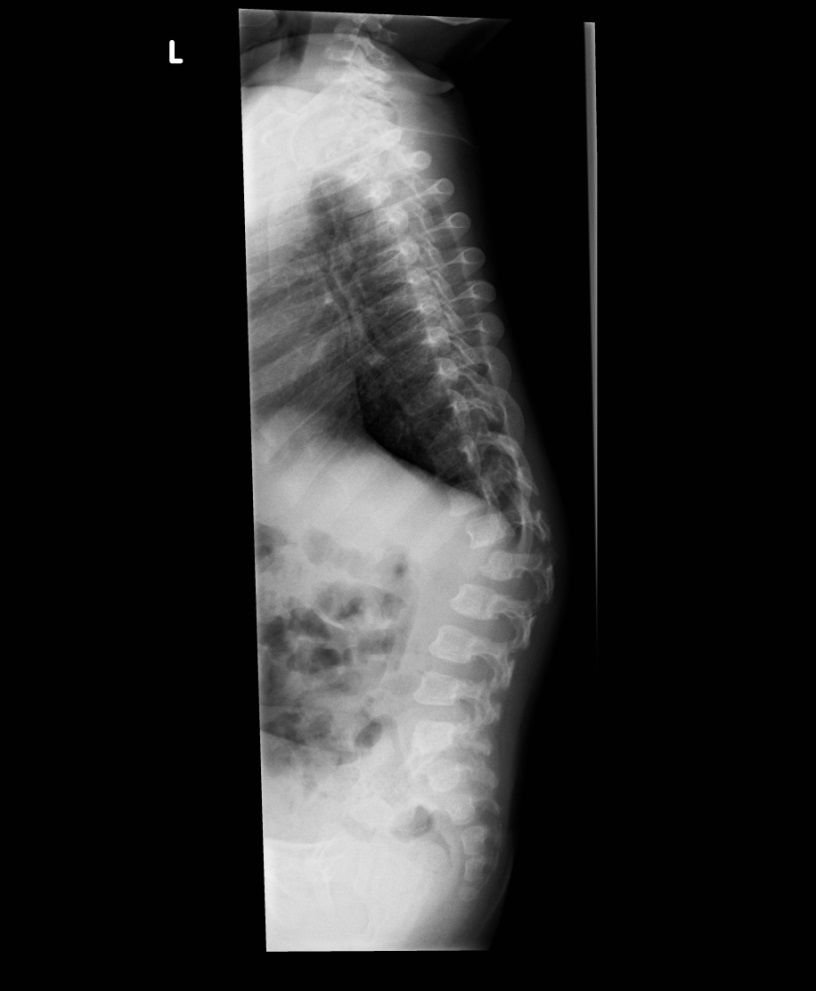

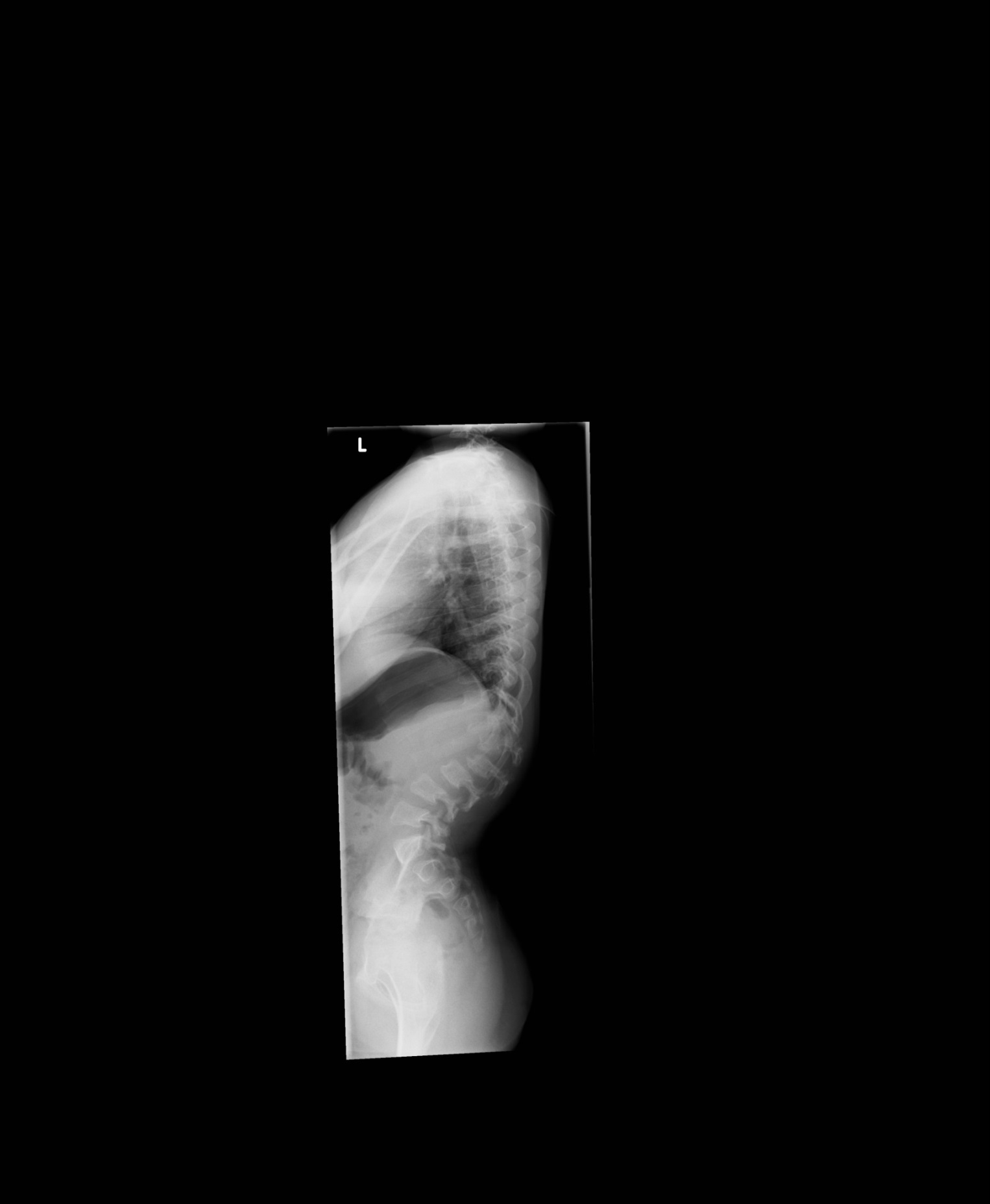

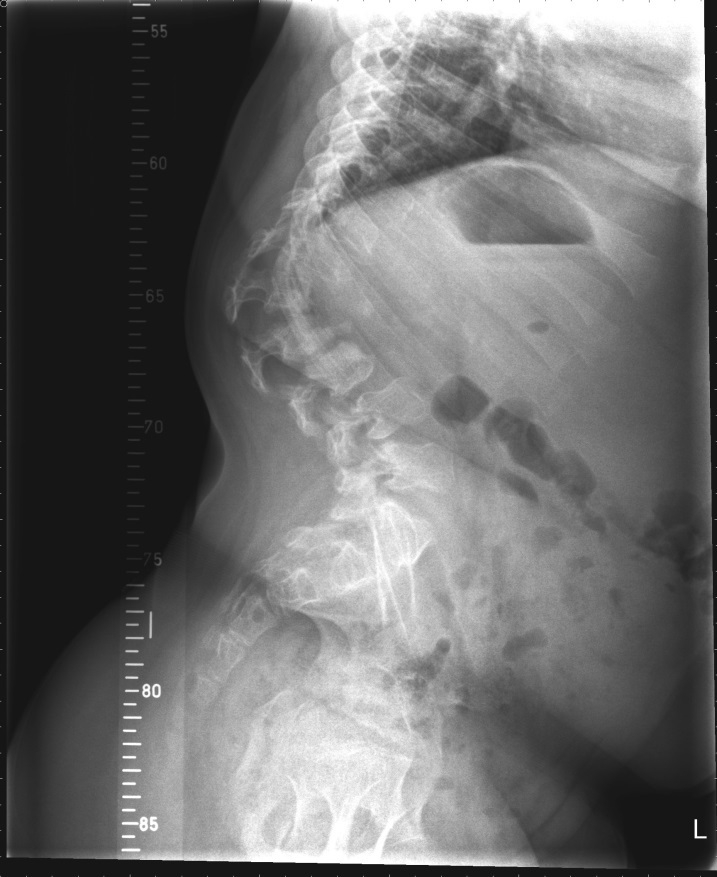


Age: 2 years
CA: 44.9°

Age: 7 years
CA: 59.9°

Age: 11.5 years

CA: 91.0°

**Questions case 1**

Please answer the following questions assuming that all patients are fit for surgery and have a sufficient quality of life.
All questions are related to MPS I patients and kyphosis surgery.

1. At the moment of the last radiograph the patient is 11.5 years of age, would you perform surgery in this patient?
   1. Yes *( go to question 2)*
   2. No *(go to question 13)*
   3. Don’t know *(go to question 13 )*
2. Would you have preferred to perform surgery earlier (before 11.5 years of age) in this patient?
   1. Yes *(go to question 3)*
   2. No *(go to question 4)*
   3. Don’t know *(go to question 4)*
3. At approximately what age? *(go to question 4)*
4. What would be the aim of the surgery? (go to question 5)
5. Does the cobb angle influence your decision about performing surgery in this patient?
   1. Yes *(go to question 6)*
   2. No *(go to question 7)*
   3. Don’t know *(go to question 7)*
6. Can you elaborate on your answer? *(go to question 7)*
7. Does progression of the cobb angle influence your decision about performing surgery in this patient?
   1. Yes *(go to question 8)*
   2. No *(go to question 9)*
   3. Don’t know *(go to question 9)*
8. Can you elaborate on your answer? *(go to question 9)*
9. Do the symptoms of this patient influence your decision about performing surgery?
   1. Yes *(go to question 10)*
   2. No *(go to question 11)*
   3. Don’t know *(go to question 11)*
10. Can you elaborate on your answer?
11. What type of surgery would you perform in this patient?
    1. Anterior spinal fusion with correction
    2. Anterior spinal fusion without correction
    3. Posterior spinal fusion with correction
    4. Posterior spinal fusion without correction
    5. Combination of anterior and posterior spinal fusion
    6. Other…
12. In your opinion, how many segments should be fused? (or: don’t know) (*go to Case 2)*

*Following question 1b and 1c*Q: Would you perform surgery in this patient?
A: No or Don't know

1. Can you elaborate on your answer please *(go to question 14)*
2. Do you need more information to decide whether surgery should be performed or not?
   1. Yes *(go to go to question 15)*
   2. No *(go to question 16)*
   3. Don’t know *(go to question 16)*
3. What information do you need? *(go to question 16)*
4. In your opinion, if you decide not to operate this child, would a brace be indicated?
   1. Yes *(go to question 17)*
   2. No *(go to question 18)*
   3. Don’t know *(go to question 18)*
5. Can you elaborate on your answer? *(go to Case 2)*

**Case 2: Patient B.**

- Male, diagnosis Hurler at 14 months
- HCT at 18 months
- Clinical course
  - Little to no progression of the cobb angle
  - Hip surgery, both sides, at age 10 years
- Current age 14 years
- Symptoms since age 11 years:
  - Bending a little bit forward, walks with support
  - No backpain
- Neurological examination: no abnormalities
- MRI: no spinal cord compression


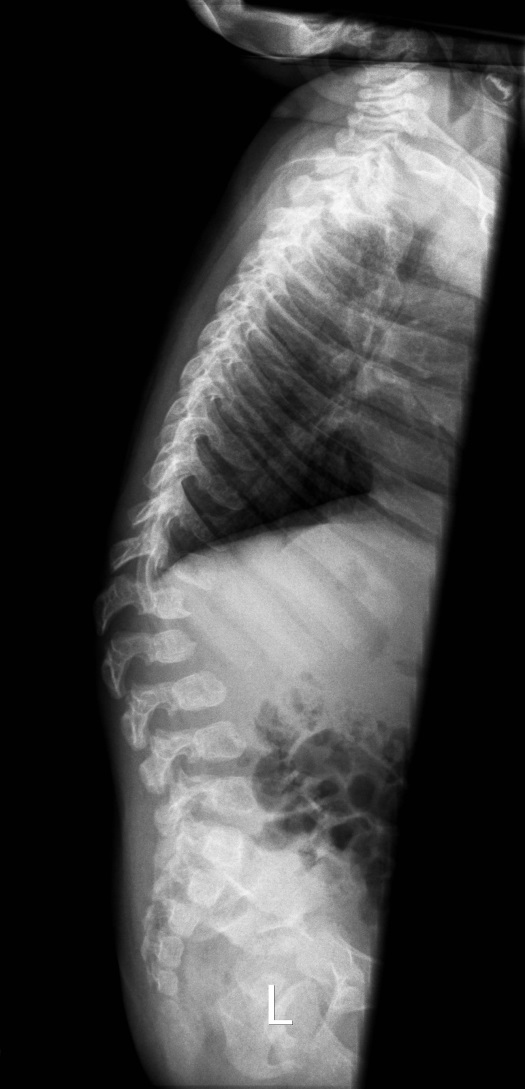

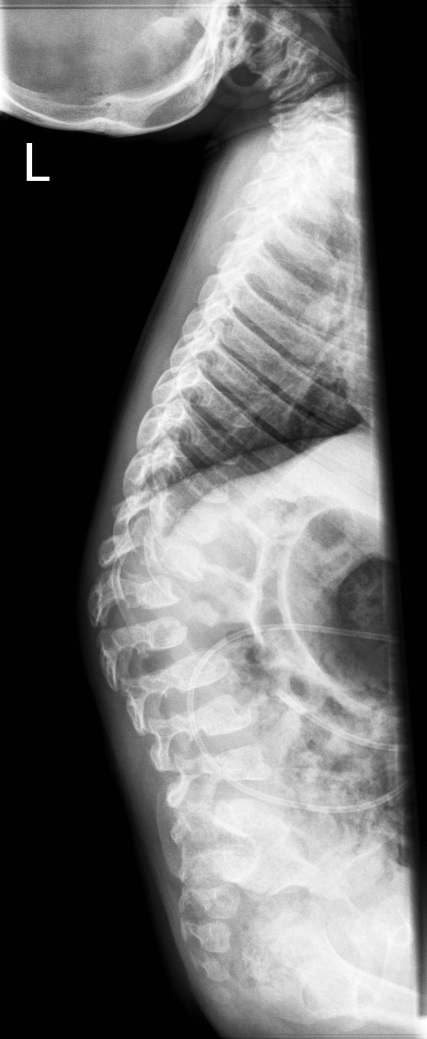

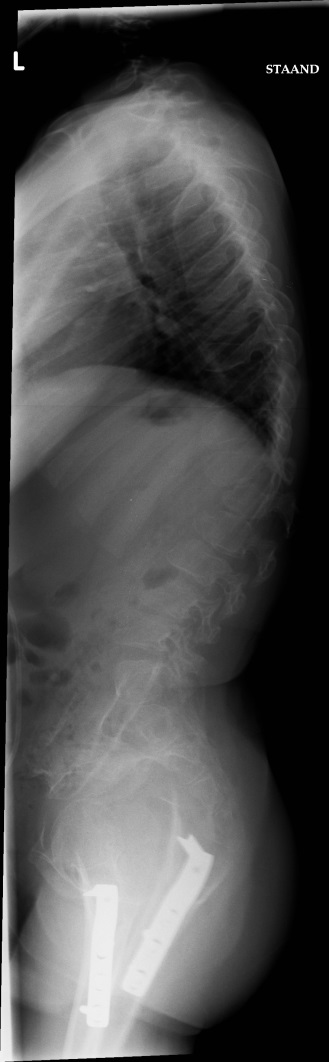


Age: 15 months

CA: 40.0°

Age: 2 yrs, 9 months

CA: 45.8°

Age: 11 yrs, 2 months

CA: 40.0°

**Questions about case 2**

Please answer the following questions assuming that all patients are fit for surgery and have a sufficient quality of life.
All questions are related to MPS I patients and kyphosis surgery.

1. At the moment of the last radiograph the patient is eleven years of age, would you now perform surgery in this patient?
   1. Yes *( go to question 19)*
   2. No *(go to question 32)*
   3. Don’t know *(go to question 32)*
2. Would you have preferred to perform surgery earlier in this patient?
   1. Yes (*go to question 20)*
   2. No *(go to question 21)*
   3. Don’t know *(go to question 21)*
3. At approximately what age? (*go to question 21)*
4. What would be the aim of the surgery? (*go to question 22)*
5. Does the cobb angle influence your decision about performing surgery in this patient?
   1. Yes *(go to question 23)*
   2. No *(go to question 24)*
   3. Don’t know *(go to question 24)*
6. Can you elaborate on your answer? *(go to question 24)*
7. Does the subluxation of Th12 influence your decision about performing surgery in this patient?*
   1. Yes *(go to question 25)*
   2. No *(go to question 26)*
   3. Don’t know *(go to question 26)*
8. Can you elaborate on your answer? please *(go to question 26)*
9. Does progression of the cobb angle influence your decision about performing kyphosis surgery in this patient?
   1. Yes *(go to question 27)*
   2. No *(go to question 28)*
   3. Don’t know *(go to question 28)*
10. Can you elaborate on your answer?
11. Do the symptoms of this patient influence your decision about performing surgery?*
    1. Yes *(go to question 29)*
    2. No *(go to section 30)*
    3. Don’t know *(go to section 30)*
12. Can you elaborate on your answer?
13. What type of surgery would you perform in this patient?
    1. Anterior spinal fusion with correction
    2. Anterior spinal fusion without correction
    3. Posterior spinal fusion with correction
    4. Posterior spinal fusion without correction
    5. Combination of anterior and posterior spinal fusion
    6. Other…
14. In your opinion, how many segments should be fused? (or: Don’t know) *(go to question 47)*

*Following question 18b and 18c*Q: At the moment of the last radiograph the patient is eleven years of age, would you now perform surgery in this patient?
A: Yes

1. Can you elaborate on your answer?
2. Do you need more information to decide whether surgery should be performed or not?
   1. Yes *(go to question 34)*
   2. No *(go to question 35)*
   3. Don’t know *(go to question 35)*
3. What information do you need? *(*go to question 35)
4. In your opinion, if you decide not to operate this child, would a brace be indicated?
   1. Yes *(go to question 36)*
   2. No *(go to question 37)*
   3. Don’t know *(go to question 37)*
5. Can you elaborate on your answer?

At the age of twelve years the cobb angle of this patient progressed from 49 to 77 degrees within one year.

1. Would you now consider surgery in this patient?
   1. Yes *(go to question 38)*
   2. No *(go to question 47)*
   3. Don’t know *(go to question 47)*
2. What would be the aim of the surgery?
3. Does the Cobb angle influence your decision about performing surgery in this patient?
   1. Yes *(go to question 40)*
   2. No *(go to question 41)*
   3. Don’t know *(go to question 41)*
4. Can you elaborate on your answer?
5. Does progression of the cobb angle influence your decision about performing kyphosis surgery in this patient?
   1. Yes *(go to question 42)*
   2. No *(go to question 43)*
   3. Don’t know *(go to question 43)*
6. Can you elaborate on your answer?
7. Do the symptoms of this patient influence your decision about performing surgery?
   1. Yes *(go to question 44)*
   2. No *(go to question 45)*
   3. Don’t know *(go to question 45)*
8. Can you elaborate on your answer?
9. What type of surgery would you perform in this patient?
   1. Anterior spinal fusion with correction
   2. Anterior spinal fusion without correction
   3. Posterior spinal fusion with correction
   4. Posterior spinal fusion without correction
   5. Combination of anterior and posterior spinal fusion
   6. Other…
10. In your opinion, how many segments should be fused? (or: Don’t know) *(go to question 47)*

**General questions – not directly related to any of the cases**

1. Is the indication for surgery primarily made by you?
   1. Yes
   2. No
2. Do you have any comments on the previous question?
3. If the cobb angle influences your decision to perform/advise surgery, from approximately what angle would you consider surgery? (or: Don’t know)
4. If progression of the cobb angle influences your decision to perform/advise surgery, what is the level of progression where you would you always consider surgery independent of the actual cobb angle?
   1. More than 10 degrees/year
   2. More than 20 degrees/year
   3. More than 30 degrees/year
   4. More than 40 degrees/year
   5. None of the above
5. Do you have any comments on the previous question?
6. To what extent do clinical symptoms contribute to your decision of performing surgery?
   1. I only look at the cobb angle
   2. I look at the cobb angle but clinical symptoms are also of some importance
   3. I look at the cobb angle but clinical symptoms are just as important
   4. In case of kyphosis, I only operate when there are clinical symptoms
   5. None of the above
7. Do you have any comments on the previous question?
8. Is the decision for kyphosis surgery based on multidisciplinary consultation?
   1. Yes *(go to question 55)*
   2. No *(go to question 56)*
9. Who is involved in your multidisciplinary team?
10. In your opinion, are there any absolute indications for kyphosis surgery in MPS I patients? (or: Don’t know)
11. In your opinion, are there any absolute contra-indications for kyphosis surgery in MPS I patients? (or: Don’t know)
12. Do you take into account the presence and severity of hip dysplasia/ the outcome of hip surgery on your decision on performing kyphosis surgery?
    1. Yes *(go to question 59)*
    2. No *(go to question 60)*
    3. Don’t know *(go to question 60)*
13. Can you elaborate on your answer?
14. Do you take into account the presence and severity of scoliosis on your decision about performing kyphosis surgery?
    1. Yes *(go to question 61)*
    2. No *(go to question 62)*
    3. Don’t know *(go to question 62)*
15. Can you elaborate on your answer?
16. Do you check for odontoid hypoplasia before performing surgery?
    1. Yes
    2. No
    3. Don’t know
17. In your opinion, is there an optimal age range for performing kyphosis surgery? (or: Don’t know)
18. Do you use braces in the treatment of kyphosis?
    1. Yes
    2. No
19. During kyphosis surgery, is neurophysiological monitoring indicated?
    1. Yes *(go to question 66)*
    2. No *(go to question 67)*
    3. Don’t know *(go to question 67)*
20. What kind of neurophysiological monitoring do you use?
    1. Somatosensory evoked potential monitoring
    2. Motor evoked potential monitoring
    3. Both
21. Do you have any additional comments on this questionnaire?
