# Supplementary material for: Treatment of thoracolumbar kyphosis in patients with mucopolysaccharidosis type I: results of an international consensus procedure
Source: Orphanet J Rare Dis. 2019 Jan 18;14:17. doi: 10.1186/s13023-019-0997-5 (PMC6339313; doi:10.1186/s13023-019-0997-5)
Supplement: Supplementary file 2 — Written round 2. (DOC 46 kb) [file 13023_2019_997_MOESM2_ESM.doc]

Additional file 2: Written round 2

The second online written round was based on information of the first written round and contained questions and statements with a focus on specific issues. The questions and statements were composed by FAW, GK and JvL.

1. The aim of kyphosis surgery in MPS I patients is prevention of ongoing progression with a satisfactory neurological, biomechanical, respiratory and cosmetic outcome for the patient.
   1. Agree
   2. Disagree
   3. Don’t know

Statement: The aim of kyphosis surgery in MPS I patients is prevention of ongoing progression with a satisfactory neurological, biomechanical, respiratory and cosmetic outcome for the patient.

1. Please elaborate on your answer.
2. The optimal age range for kyphosis surgery in MPS I patients is generally between 5 and 10 years of age.
   1. Agree *(go to question 4)*
   2. Disagree *(go to question 5)*
   3. Don’t know *(go to question 5)*
3. Why is this the optimal age range? *(go to question 6)*
4. Please elaborate on your answer.

The indication for kyphosis surgery in MPS I patients should preferably be made by a multidisciplinary team**. The spinal surgeon finalizes the decision in consensus with the patient/parents.

** Such a team preferably comprises an orthopedic surgeon, pediatrician, physiotherapist, rehabilitation specialist, neurologist, occupational therapist and anesthesiologist.

1. Statement: the indication for kyphosis surgery in MPS I patients should preferably be made by a multidisciplinary team**. The spinal surgeon finalizes the decision in consensus with the patient/parents.
   1. Agree
   2. Disagree
   3. Don’t know
2. Please elaborate on your answer.
3. Given the complexity of MPS I disease, thorough assessment of risk factors for surgery on anesthesiology, cardiopulmonary function, upper airway anatomy and odontoid hypoplasia is mandatory before kyphosis surgery in MPS I patients.
   1. Agree
   2. Disagree
   3. Don’t know
4. Please elaborate on your answer.
5. The decision to perform kyphosis surgery in MPS I patients should not only be based on the kyphotic angle, but on the combination of the angle and its progression, the expected impact on balance and the clinical symptoms.
   1. Agree
   2. Disagree
   3. Don’t know
6. Please elaborate on your answer.
7. Is myogenic back pain which is presumably caused by imbalance due to kyphosis, an indication for kyphosis surgery in MPS I patients?
   1. Yes
   2. No
   3. Don’t know
8. Please elaborate on your answer?
9. Neurological signs and symptoms caused by kyphosis are an indication for kyphosis surgery.
   1. Agree
   2. Disagree
   3. Don’t know
10. Please elaborate on your answer.
11. The developmental quotient does NOT play a role in the decision for kyphosis surgery.
    1. Agree
    2. Disagree
    3. Don’t know
12. Please elaborate on your answer.
13. Expected quality of life has a key role in the decision for kyphosis surgery.
    1. Agree
    2. Disagree
    3. Don’t know
14. Please elaborate on your answer.
15. The presence and severity of hip dysplasia should be taken into account when kyphosis surgery is considered.
    1. Agree
    2. Disagree
    3. Don’t know
16. Please elaborate on your answer.
17. In the presence of hip dysplasia and a kyphosis, hip surgery should preferably be performed before kyphosis surgery.
    1. Agree
    2. Disagree
    3. Don´t know
18. Please elaborate on your answer.
19. The following should be assessed on the spinal X-ray when considering kyphosis surgery: (please tick one box in each row)
    1. Degree of listhesis
       1. Yes
       2. No
       3. Don’t know
    2. Sagittal offset
       1. Yes
       2. No
       3. Don’t know

For listhesis, if yes;

1. How would the degree of listhesis influence your decision making on kyphosis surgery?

For sagittal offset, if yes;

1. How would the outcome of sagittal offset influence your decision making on kyphosis surgery?
2. Measuring the kyphotic angle in MPS I patients can be difficult due to dysplasia of the vertebra. Should there be a standardized protocol stating how to measure the kyphotic angle in these patients?
   1. Yes
   2. No
   3. Don’t know
3. Patient positioning for spinal X-ray should be standardized (preferably standing, unsupported). If this is not feasible for an individual patient it should be reported in the conclusion of the report.
   1. Agree
   2. Disagree
   3. Don’t know
4. Sagittal offset should be taken into account when kyphosis surgery in MPS I patients is considered. What is the optimal method for measuring sagittal offset in these patients?
5. Neurological monitoring is indicated during kyphosis surgery. This should preferably be combined somatosensory evoked potential monitoring and motor evoked potential monitoring.
   1. Agree
   2. Disagree
   3. Don’t know

Statement: Neurological monitoring is indicated during kyphosis surgery. This should preferably be combined somatosensory evoked potential monitoring and motor evoked potential monitoring.

1. Please elaborate on your answer.
2. In your opinion, what are the benefits of the posterior only approach for kyphosis surgery?
3. In your opinion, what are the cons of the posterior only approach for kyphosis surgery?
4. In your opinion, what are the benefits of the combined approach?
5. In your opinion, what are the cons of the combined approach?
6. What are the items that play a role in the choice of type of surgery (posterior only or combined approach)?
   1. Age
      1. Yes
      2. No
      3. Don’t know
   2. Angle
      1. Yes
      2. No
      3. Don’t know
   3. Progression
      1. Yes
      2. No
      3. Don’t know
7. Please elaborate on the answers of the previous question, e.g. are there any other items that play a role in the choice of the surgical approach?
8. How do you decide on the number of segments to be fused?
9. Do you advice braces in kyphosis treatment prior to surgery?
   1. Yes
   2. No
   3. Don’t know
10. Please elaborate on your answer.
11. Do you advice braces in kyphosis treatment post-surgery?
    1. Yes
    2. No
    3. Don’t know
12. Please elaborate on your answer.
13. Are there any statements that you would like to add?

**Vignette 1**

Male patient, current age 7 years

- Hurler, diagnosed at age 2 years
- HCT at age 2,5 years
- Hip surgery at 5 years of age
- Normal cognition
- Evaluated by pulmonologist, cardiologist, anesthesiologist and fit for surgery
- No neurological impairment
- No pain
- Good quality of life

What would be a likely scenario in which you would consider surgery in this patient after one year of follow- up?

46. From what angle progression would you consider surgery in this patient after one year of follow-up?

**Vignette 2**

Male patient, current age 7 years

- Hurler, diagnosed at age 2 years
- HCT at age 2,5 years
- Hip surgery at 5 years of age
- Normal cognition
- Evaluated by pulmonologist, cardiologist, anesthesiologist and fit for surgery
- No neurological impairment
- HAS BACK PAIN (in contrast to vignette 1)
- Good quality of life

What would be a likely scenario in which you would consider surgery in this patient after one year of follow-up?

47. From what angle progression would you consider surgery in this patient after one year of follow-up?

48. Do you have any additional comments on this questionnaire?
