# Supplementary material for: Treatment of thoracolumbar kyphosis in patients with mucopolysaccharidosis type I: results of an international consensus procedure
Source: Orphanet J Rare Dis. 2019 Jan 18;14:17. doi: 10.1186/s13023-019-0997-5 (PMC6339313; doi:10.1186/s13023-019-0997-5)
Supplement: Supplementary file 3 — Draft statements to be discussed at the face-to-face meeting. (DOC 67 kb) [file 13023_2019_997_MOESM3_ESM.doc]

Additional file 3: Draft statements to be discussed at the face-to-face meeting

The draft statements were composed by FAW, GK and JvL and presented and discussed at the face-to-face meeting.

**STATEMENT 1 - DRAFT**The aim of kyphosis surgery in MPS I patients is prevention of ongoing progression of kyphosis with a satisfactory neurological, biomechanical (i.e. improvement of sagittal balance), respiratory and cosmetic outcome for the patient, aimed at improvement of daily life performance.

**STATEMENT 2 - DRAFT**
The timing of surgery depends on kyphosis progression and on the presence of symptoms, but also on the flexibility of the spinal curve, the presence of post HCT complications and growth potential. In general, surgery will best be done approximately between 5 and 13 years of age.

**STATEMENT 3 – DRAFT**Acknowledging that the spinal surgeon, together with patients/parents will make the decision for kyphosis surgery in MPS I patients, consultation of a multidisciplinary team* should preferably precede this decision.

**STATEMENT 4 – DRAFT**
Given the complexity of MPS I disease, thorough assessment of risk factors for surgery on anesthesiology, cardiopulmonary function, upper airway anatomy and odontoid hypoplasia is mandatory before kyphosis surgery in MPS I patients.

**STATEMENT 5 = DRAFT - ADDITIONAL STATEMENT** (proposed by a participant during written round 2) Preoperative spine MRI should be evaluated for spinal cord compression at sites away from the gibbus deformity, particularly the occipital-cervical junction and the cervico-thoracic junction.

**STATEMENT 6 – DRAFT**
The decision to perform kyphosis surgery in MPS I patients should not only be based on the kyphotic angle, but on the combination of the angle, its progression and the expected impact of surgery on (future) impairments and activities.

**STATEMENT 7 – DRAFT**Myogenic back pain as a single symptom is not a reason to perform surgery. It is essential to first determine the cause of the pain and explore other therapeutic options (e.g. physiotherapy, pain medication, brace). Surgery can be considered after all other options to treat the pain have failed.

**STATEMENT 8 – DRAFT**Neurological signs and symptoms caused by kyphosis (though presumably rare) are an indication for kyphosis surgery.

**STATEMENT 9 – DRAFT**The developmental quotient does NOT play a role in the decision for kyphosis surgery in general. However, if no improvement in activities, daily functioning and participation can be expected, one may decide to abstain from surgery.
 **STATEMENT 10 – DRAFT**Expected quality of life (as determined by body functions, activities and participation*) has a pivotal role in the decision for kyphosis surgery. *ICF

**STATEMENT 11 – DRAFT**Although hip dysplasia and kyphosis are independent problems (both caused by dysostosis multiplex), they both influence the sagittal balance. Therefore the presence and severity of hip dysplasia should be taken into account when kyphosis surgery is considered.
 **STATEMENT 12 - DRAFT**
Timing of hip surgery and kyphosis surgery in a patient needs to be assessed by a hip surgeon and spinal surgeon (and in consultation with a multidisciplinary team) as they both impact balance. However in general, hip surgery will be performed at relatively earlier age because that allows reconstructive surgery while at an older age a salvage procedure will be the only option.
 **STATEMENT 13 – DRAFT**
Patient positioning for spinal X-ray should be standardized (preferably standing, unsupported). If this is not feasible for an individual patient it should be reported in the conclusion of the report.

**STATEMENT 14 – DRAFT**There should be a standardized protocol stating how to measure the kyphotic angle in patients with MPS I.

**STATEMENT 15 – DRAFT**Listhesis should be assessed on the spinal X-ray when considering kyphosis surgery.

**STATEMENT 16 – DRAFT**There should be a standardized protocol stating how to measure the spondylolisthesis in patients with MPS I.

**STATEMENT 17 – DRAFT**
Sagittal offset should be assessed on the spinal X-ray when considering kyphosis surgery.

**STATEMENT 18 – DRAFT**There should be a standardized protocol stating how to measure the sagittal offset in patients with MPS I.

**STATEMENT 19 – DRAFT**Neurological monitoring is indicated during kyphosis surgery. This should preferably be combined somatosensory evoked potential monitoring and motor evoked potential monitoring.
 **STATEMENT 20 – DRAFT**The posterior only approach and the combined approach both have benefits and disadvantages, and the decision for the approach should be based on the patient’s age and the degree of deformity, stiffness and bone dysplasia.

**STATEMENT 21 – DRAFT**During the face-to-face meeting two surgeon were asked to develop a statement on the number of segments to be fused.

**STATEMENT 22- DRAFT**

Bracing prior to surgery may be considered as it may postpone surgery, however acceptability of a brace by children may be very low.

**STATEMENT 23 – DRAFT**Bracing post-surgery may be considered for a period of 3 to 6 months as it may protect the arthrodesis and the adjacent segments and also stabilizes the spine. However acceptability of a brace by children may be very low.

**STATEMENT 24 – DRAFT**When kyphosis surgery is considered, the extent of the angle progression/year is less important when the initial angle is bigger
